# SspH, a Novel HATPase Family Regulator, Controls Antibiotic Biosynthesis in *Streptomyces*

**DOI:** 10.3390/antibiotics11050538

**Published:** 2022-04-19

**Authors:** Xue Yang, Yanyan Zhang, Shanshan Li, Lan Ye, Xiangjing Wang, Wensheng Xiang

**Affiliations:** 1 State Key Laboratory for Biology of Plant Diseases and Insect Pests, Institute of Plant Protection, Chinese Academy of Agricultural Sciences, Haidian District, Beijing 100193, China; yangxue504499816@163.com (X.Y.); ssli@ippcaas.cn (S.L.); yelan_617@163.com (L.Y.); 2 School of Life Science, Northeast Agricultural University, Harbin 150030, China; wangneau2013@163.com

**Keywords:** global regulator, milbemycins, *Streptomyces bingchenggensis*, SspH, actinorhodin, avermectin

## Abstract

*Streptomyces* can produce a wealth of pharmaceutically valuable antibiotics and other bioactive compounds. Production of most antibiotics is generally low due to the rigorously controlled regulatory networks, in which global/pleiotropic and cluster-situated regulatory proteins coordinate with various intra- and extracellular signals. Thus, mining new antibiotic regulatory proteins, particularly the ones that are widespread, is essential for understanding the regulation of antibiotic biosynthesis. Here, in the biopesticide milbemycin producing strain *Streptomyces bingchenggensis*, a novel global/pleiotropic regulatory protein, SspH, a single domain protein containing only the HATPase domain, was identified as being involved in controlling antibiotic biosynthesis. The *sspH* overexpression inhibited milbemycin production by repressing the expression of milbemycin biosynthetic genes. The *sspH* overexpression also differentially influenced the expression of various antibiotic biosynthetic core genes. Site-directed mutagenesis revealed that the HATPase domain was essential for SspH’s function, and mutation of the conserved amino acid residues N54A and D84A led to the loss of SspH function. Moreover, cross-overexpression experiments showed that SspH and its orthologs, SCO1241 from *Streptomyces coelicolor* and SAVERM_07097 from *Streptomyces avermitilis*, shared identical functionality, and all exerted a positive effect on actinorhodin production but a negative effect on avermectin production, indicating that SspH-mediated differential control of antibiotic biosynthesis may be widespread in *Streptomyces*. This study extended our understanding of the regulatory network of antibiotic biosynthesis and provided effective targets for future antibiotic discovery and overproduction.

## 1. Introduction

*Streptomyces* can produce numerous pharmaceutically valuable antibiotics, many of which have been widely used in veterinary, agricultural and medical areas. On the other hand, sequencing of *Streptomyces* genomes also revealed the existence of a large number of cryptic and uncharacterized antibiotic biosynthetic gene clusters (BGCs) other than the known antibiotic BGCs [1]. The expression of antibiotic BGCs is rigorously controlled by pyramidal regulatory cascades, in which global/pleiotropic and cluster-situated transcriptional regulatory proteins from different families coordinate with environmental and physiological cues [2,3,4]. Meanwhile, this rigorous control plays a key role in determining the production level of antibiotics under specific fermentation conditions. To date, many different families of regulatory proteins have been identified and their role in the biosynthesis of antibiotics have been characterized in depth, thus generating regulator-based strategies—such as activator overexpression, repressor deletion, promoter replacement, BGC refactoring and duplication—to achieve antibiotic overproduction and activation [5,6]. However, there still exist a large number of transcriptional regulatory factors with unknown functions in the genome of *Streptomyces*, and our understanding of the antibiotic production regulatory network is very poor; discovering new regulators, particularly the highly conserved ones, is necessary, and will enrich our knowledge of the antibiotic regulatory network and provide new strategies for antibiotic overproduction and activation.

In the natural world, bacteria live in diverse environments and are frequently exposed to nutrient scarcity and challenging environmental conditions. To survive in such unfavorable conditions, they have evolved complex adaptive response systems including the two-component system (TCS). *Streptomyces* possesses a large number of TCSs to cope with complicated, changing and competitive environments, and many TCSs have been shown to impact morphological development and (or) antibiotic production under laboratory culture conditions [7,8]. A typical TCS consists of a membrane-bound histidine kinase (HK) and a cytoplasmic response regulator (RR). In response to a specific signal, the HK autophosphorylates at a conserved His residue, and the phosphoryl group is subsequently transferred to an Asp residue of the RR; the RR is then activated and interacts with target promoter regions to switch on the downstream cell responses [9]. Generally, a typical HK is mainly composed of the periplasmic sensing domain, the dimerization domain (also known as the HK acceptor [HisKA] domain) and the HK-like ATPase (HATPase) domain. The HisKA domain possesses the conserved histidine residue that can be phosphorylated by the HK itself, and the HATPase domain is characterized by five conserved amino acid motifs (N, G1, F, G2 and G3 boxes) [10,11]. Due to the presence of the HATPase domain, the HK also belongs to the GHKL (Gyrase, Hsp90, HK and MutL) ATPase/Kinase superfamily, and the conserved F Motif is unique to HK, distinguishing the HK from the other members of the GHKL family. Interestingly, bioinformatics analysis of the *Streptomyces* genomes revealed the existence of a unique class of one-component signal transduction systems, which shared high similarity with the HK, but contained only the HATPase domain and did not contain the HisKA autophosphorylation site (here, these single domain proteins with only the HATPase are named HATPase-ol proteins). For example, there are at least 26 HATPase-ol proteins in the model strain *Streptomyces coelicolor*, 24 in the avermectin producing strain *Streptomyces avermitilis* and 21 in the milbemycin producing strain *Streptomyces bingchenggensis* (Supplementary Table S1), indicating that HATPase-ol proteins may be widespread in *Streptomyces*; however, little is known about their biological functions.

Polyketide milbemycins are a group of 16-membered macrolide chemicals. The main active components, milbemycin A3/A4 and the derivatives of milbemycin A3/A4 (lepimectin, latidectin and milbemycin oxime), have been commercialized as biopesticides and veterinary drugs [12]. *S. bingchenggensis* is an important industrial producer of milbemycins. Its genome contains hundreds of regulatory genes, but the functions of only a few of them are known, including the milbemycin cluster-situated regulators MilR and MilR2 [12,13], the quorum-sensing system SbbR/SbbA [14], the pleiotropic SARP family regulator KelR [15], and the four-component system SbrH1-R [16]. Thus, our knowledge of the regulatory network of milbemycin biosynthesis is still limited; mining novel transcriptional regulators affecting milbemycin production is necessary to improve the overall understanding of the milbemycin biosynthesis regulatory network and will contribute to the full exploitation of potential targets influencing milbemycin production.

In this report, we discovered a novel regulator of milbemycin biosynthesis, SBI_08867 (also named *S.* *bingchenggensis* small protein containing only the HATPase domain [SspH]). SspH consists of 142 amino acid residues and contains only the HATPase domain, thus it belongs to the HATPase-ol subfamily. We demonstrated that SspH may control milbemycin production by repressing the expression of milbemycin BGC. SspH also played differential roles in the control of multiple other antibiotic BGCs present in the *S. bingchenggensis* genome. SspH and its orthologs (SCO1241 and SAVERM_07097) shared the same function; both were positive regulators of actinorhodin biosynthesis in *S. coelicolor* and negative regulators of avermectin production in *S. avermitilis*. These results enriched our understanding of the antibiotic regulatory network and provided a useful method for antibiotic activation and overproduction.

## 2. Results

### 2.1. Identification of the Novel HATPase-ol Family Regulator SspH in S. bingchenggensis

**Figure 1:**
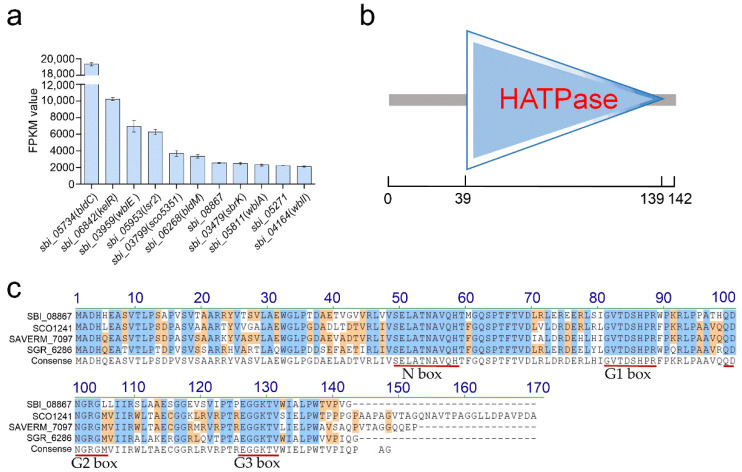
Identification of SBI_08867 in *S. bingchenggensis*. (**a**) Transcript abundances of genes with FPKM values higher than 2000 measured by RNA-seq on day 3. (**b**) Predicted domain structure of SBI_08867. HATPase, histidine kinase-like ATPase. (**c**) Amino acid sequence alignment of SBI_08867 and its orthologs. Identical or similar residues in all sequences are highlighted in blue and orange, respectively. The corresponding assumed conserved motifs are marked in red. SCO1241, SBI_08867 ortholog from *S. coelicolor*; SAVERM_7097, SBI_08867 ortholog from *S. avermitilis*; SGR_6286, SBI_08867 ortholog from *S. griseus*.

In our recent work, the two hypothetical proteins (SbrH1H2) and the typical TCS (SbrKR) that together comprise the four-component system SbrH1-R (also known as SbrH1/H2/K/R) have been identified to be negative regulators of milbemycin biosynthesis. SbrH1H2 and SbrKR act synergistically to repress milbemycin biosynthesis [16]. Remarkably, of all the detected genes, the FPKM values of *sbrH1* and *sbrH2* were the highest during the entire milbemycin production period, suggesting that regulatory factors with relatively high expression may play a role in milbemycin biosynthesis. Based on this speculation, we extracted regulatory genes with FPKM values higher than 2000 from the transcriptomic data of *S. bingchenggensis* BC04 collected on day 3 (when milbemycin production begins) to mine new regulators influencing milbemycin production. A total of eleven candidate transcriptional regulatory genes were obtained (Figure 1a), of which two (*kelR* and *sbrK*) have been shown to be involved in milbemycin biosynthesis [15,16], eight (e.g., *sbi_05734*, *sbi_03959*, *sbi_05953*, *sbi_03799*, *sbi_06268*, *sbi_08867*, *sbi_05811* and *sbi_04164*) may be widespread in *Streptomyces* and even actinobacteria (KEGG homolog analysis), and one (*sbi_05271*) encodes a MarR family regulator whose homologs are only found in several *Streptomyces* species (Table 1 and Table S2). Among the eight widespread regulatory genes, orthologs of seven genes have been reported in other *Streptomyces* species to be involved in developmental differentiation and/or antibiotic biosynthesis [17,18,19,20,21,22,23], but nothing is known about the function of *sbi_08867* and its orthologs. The *sbi_08867* gene encodes a 15.2-kDa polypeptide containing only the HATPase domain (amino acids 39–139) (SMART No. SM000387) (Figure 1b), which is present in several functionally diverse proteins including the gyrase class of DNA topoisomerases, the heat shock protein HSP90, HK and MutL-like DNA mismatch repair proteins (collectively known as GHKL superfamily proteins) [10]. The HATPase domain contains four conserved amino acid motifs, namely, N, G1, G2 and G3 boxes, which form the nucleotide binding site [11]. As for SBI_08867 and its orthologs, the corresponding assumed conserved motifs are shown in Figure 1c. Furthermore, PSI-BLAST was used to search the nonredundant protein sequence (nr) database for SBI_08867 homologs (query coverage 90% to 100% and percent identity 60% to 100%). A total of 1395 hits were obtained, and almost 99% of the hits were from *Streptomyces*, indicating that SBI_08867 and its homologs were mainly present in *Streptomyces* species (Table S3). Thus, determining the roles of SBI_08867 (hereafter named SspH) and its homologs is necessary, strengthening our knowledge about *Streptomyces* genetics.

### 2.2. SspH Negatively Influences Milbemycin Production

**Figure 2:**
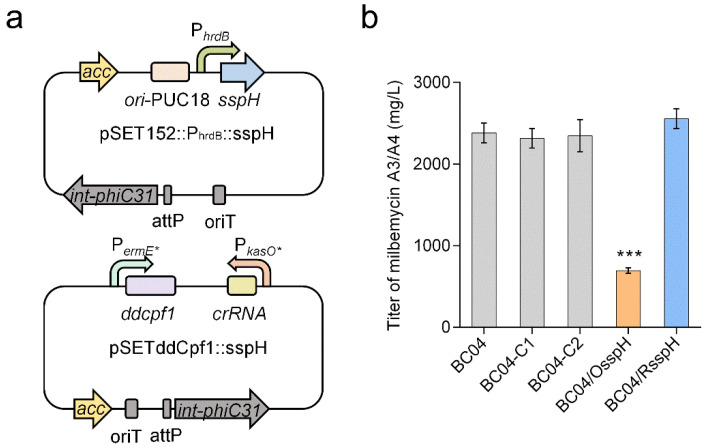
Effects of *sspH* overexpression and repression on milbemycin production. (**a**) Schematic diagrams of *sspH* overexpression and repression plasmids. The *hrdB* promoter P*_hrdB_* was used to drive the expression of *sspH*. Two strong promoters, P_ermE*_ and P_kasO*_, were used to drive the expression of *ddcpf1* and *crRNA*, respectively. (**b**) Titer of milbemycin A3/A4 in strains BC04, BC04-C1, BC04-C2, BC04/OsspH and BC04/RsspH. The milbemycin titer was determined after fermentation for 9 days. BC04, the parental strain; BC04-C1, BC04 with pSET152::P_hrdB_; BC04-C2, BC04 with pSETddCpf1; BC04/OsspH, *sspH* overexpression strain; BC04/RsspH, *sspH* repression strain. Data are presented as the averages of three independent experiments conducted in triplicate. Error bars show standard deviations from three replicates. *p*-values were determined by Student’s *t*-test. *** *p* < 0.001.

To determine whether SspH affected the biosynthesis of milbemycins, an *sspH* overexpression strain (BC04/OsspH) and repression strain (BC04/RsspH) were constructed using pSET152::P_hrdB_ (with this plasmid, target gene overexpression is controlled by the *hrdB* promoter) and the ddCpf1-based CRISPRi system pSETddCpf1, respectively (Figure 2a). Moreover, pSET152::P_hrdB_ and pSETddCpf1 were also introduced into BC04 to obtain control strains BC04-C1 and BC04-C2, respectively. Then the four strains and the parental strain BC04 were cultured and tested for milbemycin A3/A4 production. Compared with BC04 and the control strains (BC04-C1 and BC04-C2), BC04/OsspH showed an obvious decrease in milbemycin production while BC04/RsspH showed a slight increase in milbemycin titer (Figure 2b), indicating that SspH played a negative role in milbemycin production.

### 2.3. SspH Represses the Transcription of milR, milF and milA1

**Figure 3:**
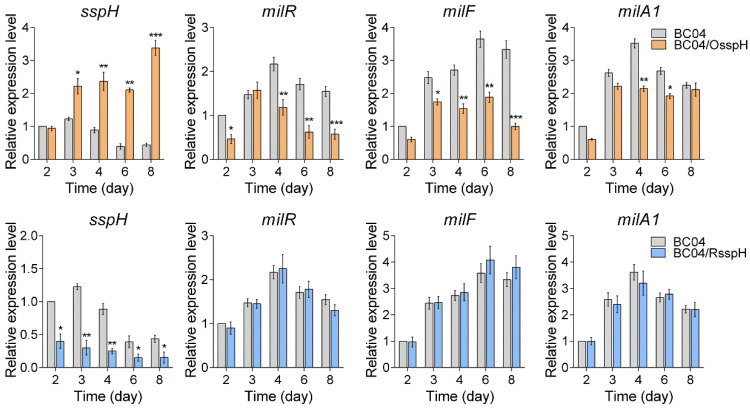
The qRT-PCR analysis of *sspH*, *milR*, *milF* and *milA1* in BC04, BC04/OsspH and BC04/RsspH. The transcriptional levels of related genes are presented relative to that of BC04 on day 2. 16S rRNA was used as the internal control. Data are presented as the averages of three triplicates. Error bars show standard deviations. * *p* < 0.05, ** *p* < 0.01, *** *p* < 0.001.

To determine the effects of SspH on the expression of milbemycin BGC genes, qRT-PCR analysis was carried out to assess the transcriptional levels of *milR* (the milbemycin cluster-situated activator gene) [12], *milF* (the tailoring enzyme gene controlled by MilR) [24] and *milA1* (type I polyketide synthase gene) [25] (Table S4). Total RNAs were extracted from the mycelia of BC04, BC04/OsspH and BC04/RsspH cultivated for various days (2, 3, 4, 6 and 8 days). Transcription of *sspH* in BC04 peaked on day 3 (when milbemycin production began) and decreased (but remained relatively high) thereafter. This transcriptional profile was similar to that of *milR*. As expected, the transcript level of *sspH* was significantly higher in BC04/OsspH than in BC04 at most time points, suggesting the successful overexpression of *sspH* (Figure 3). In contrast, transcription of *milR* was obviously lower in BC04/OsspH compared with BC04 at most time points. Similar results were also observed for *milF* and *milA1* (Figure 3), suggesting that SspH may control milbemycin production by repressing milbemycin biosynthetic genes. In BC04/RsspH, the expression level of *sspH* decreased obviously and was only 30–40% of that in BC04, indicating a partial inhibition by the CRISPRi system. However, no significant change was observed for *milR* and *milA1*, and only *milF* showed a little increase in BC04/RsspH at the middle and late fermentation stages (6 to 8 days) (Figure 3). Perhaps it is not surprising that the titer of milbemycins and the expression of milbemycin BGC showed no significant change, because although the transcript level of *sspH* was significantly downregulated, the resultant transcript level was still very high and could effectively inhibit the production of milbemycins.

### 2.4. The sspH Overexpression in BC04 Differentially Affects Expression of Multiple PKS/NRPS/PKS-NRPS Genes

**Figure 4:**
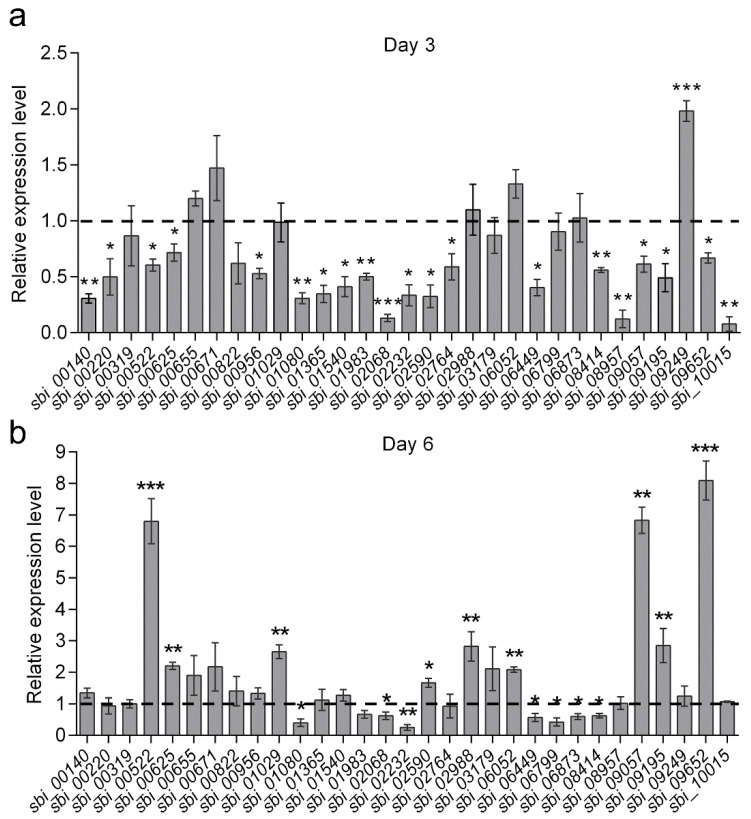
The qRT-PCR analysis of 31 PKS, NRPS and PKS-NRPS biosynthetic core genes in BC04 and BC04/OsspH (**a**) on day 3 and (**b**) on day 6. RNA samples were isolated from 3- and 6-day cultures. The expression level of each biosynthetic core gene in BC04 was assigned a value of 1 (represented by the dotted line). 16S rRNA was used as the internal control. Data are presented as the averages of three triplicates. Error bars show standard deviations. * *p* < 0.05, ** *p* < 0.01, *** *p* < 0.001.

PKS, NRPS and PKS-NRPS genes are the three main types of antibiotic BGCs within the genome of *Streptomyces*. In *S. bingchenggensis*, there are about 31 such gene clusters. To determine whether *sspH* overexpression affected the expression of PKS/NRPS/PKS-NRPS genes, the transcriptional levels of 31 biosynthetic core genes from these PKS/NRPS/PKS-NRPS clusters were detected by qRT-PCR in strains BC04 and BC04/OsspH (Table S5). Transcription of most biosynthetic core genes was downregulated, and only one gene (*sbi_09249*) showed about 2-fold upregulation in BC04/OsspH on day 3 (Figure 4a). In BC04/OsspH on day 6, eight genes (*sbi_00522*, *sbi_00625*, *sbi_01029*, *sbi_02988*, *sbi_06052*, *sbi_09195*, *sbi_09057* and *sbi_09652*) showed 2- to 6-fold upregulation (Figure 4b), while the other genes exhibited similar or lower expression levels than in BC04. These results suggested that SspH may be a pleiotropic regulator differentially influencing the expression of multiple antibiotic BGCs, and *sspH* overexpression was effective in upregulating some cryptic and uncharacterized antibiotic BGCs.

### 2.5. The Conserved HATPase Domain Is Essential for SspH Function

SspH is a single domain protein containing only the HATPase domain. To verify the importance of the HATPase domain, we intended to perform site-directed mutation of certain conserved amino acid residues. Among the four conserved binding motifs, the Asn residue (essential for chelating Mg^2+^) from the N box and the Asp residue (which interacts directly with ATP and forms a hydrogen bond with an adenine base) from the G1 box are absolutely conserved in HATPase domain containing proteins [10,26], so we decided to perform point mutations of these two key residues. First we identified the two conserved amino acids from the SspH amino acid sequence. As for SspH, the conserved Asn residue of the N box should be Asn54. This is easy to determine because SspH’s N box shares the core sequence “NA” with the N box of the HK HATPase domain. However, the G1 box of the HATPase domain of SspH seemed to have a unique characteristic that distinguished it from the HK and most other members of the GHKL family. Secondary and three-dimensional (3D) structure analysis of SspH showed that the Asp84 residue at the C-terminal end of sheet β3 might be the conserved Asp residue of the G1 motif that could directly interact with ATP, but its downstream flanking sequence (the loop region between sheet β3 and helix α3) did not contain Gly (Figure S1), which was different from the G1 motif core sequence (core sequence: DxGxG) of most GHKL family members; instead, a Gly81 upstream of Asp84 was found within the β3 sheet (Figure S1). We speculate that this arrangement of Gly81 and Asp84 might be a unique feature of the G1 motif of SspH-like proteins. To determine whether this arrangement of Asp and Gly in the G1 motif was conserved among SspH-like proteins, the G1 motifs of nine orthologs of SspH were analyzed. As expected, the relative positions of the conserved Asp and Gly in the G1 motifs of these proteins were the same as in the G1 motif of SspH (Figure S2).

**Figure 5:**
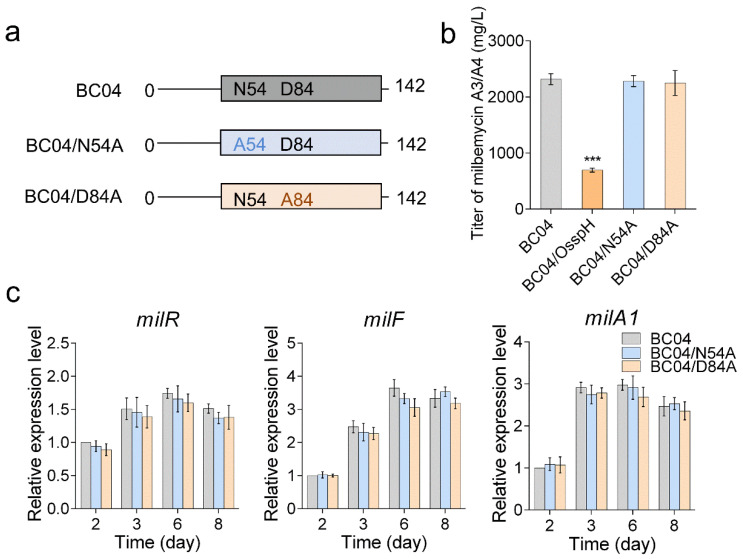
Effects of site-directed mutagenesis in the HATPase domain of SspH on its function. (**a**) Diagrams of site-directed mutation in the HATPase domain. A54, Asn54 was mutated to Ala; A84, Asp84 was changed to Ala. (**b**) Titers of milbemycin A3/A4 in strains BC04, BC04/OsspH, BC04/N54A and BC04/D84A. (**c**) qRT-PCR analysis of the transcriptional levels of *milR*, *milF* and *milA1* in BC04, BC04/N54A and BC04/D84A. The expression of each gene in BC04 on day 2 was assigned a value of 1. The *p*-values were determined by Student’s *t*-test. *** *p* < 0.001.

Next, after the conserved Asn54 and Asp84 in SspH were identified, the two sites were mutated to alanine to verify their importance (Figure 5a). Two mutagenized *sspH* plasmids pSET152::N54A and pSET152::D84A were constructed and further introduced into BC04 to generate strains BC04/N54A and BC04/D84A. The two strains, together with BC04 and BC04/OsspH, were compared for milbemycin production. The results showed that the titers of milbemycin A3/A4 in the two *sspH* mutant overexpression strains were similar to that in BC04 (Figure 5b). To determine the influence of the mutant *sspHs* on the expression of the milbemycin BGC, qRT-PCR was performed again to detect expression changes of *milR*, *milF* and *milA1*. As expected, the expression levels of the three genes in BC04/N54A and BC04/D84A were comparable to those in BC04 (Figure 5c). These results strongly suggested that Asn54 and Asp84 were the key active residues and that the HATPase domain was essential for the function of SspH.

### 2.6. SspH Is Commonly Involved in the Control of Antibiotic Production in Streptomyces

**Figure 6:**
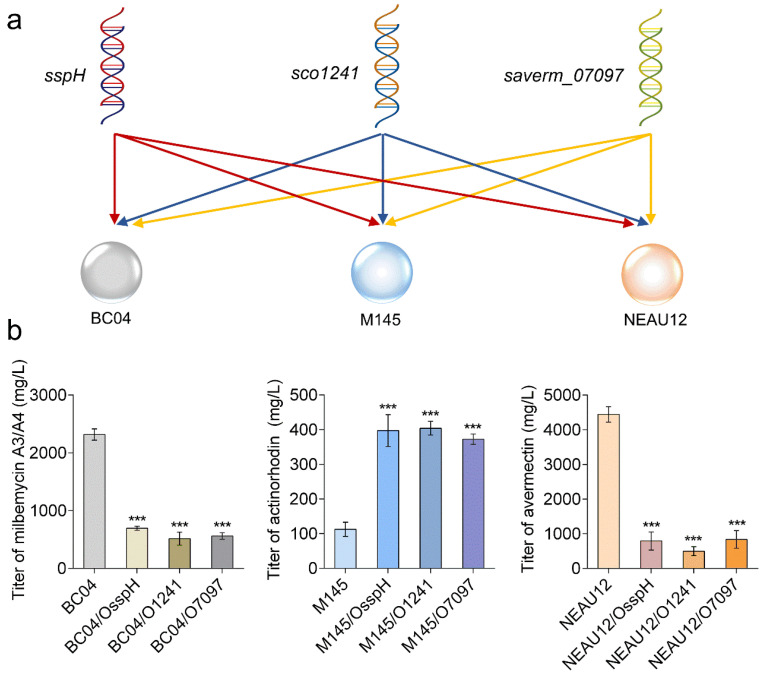
SspH and its orthologs are involved in modulating antibiotic production in three *Streptomyces* species. (**a**) Schematic diagrams of cross-overexpression of *sspH*, *sco1241* and *saverm_07097* in BC04, M145 and NEAU12. (**b**) Effects of overexpression of *sspH*, *sco1241* and *saverm_07097* on the titers of milbemycin A3/A4, actinorhodin and avermectin B1a in BC04, M145 and NEAU12. *p*-values were determined by Student’s *t*-test. *** *p* < 0.001.

As mentioned above, SspH homologs are widely distributed in *Streptomyces*. To assess whether SspH and its homologs were involved in modulating antibiotic production in other *Streptomyces* species, gene overexpression tests were also performed in the model strain *S. coelicolor* M145 and in the avermectin industrial producer *S. avermitilis* NEAU12 (Figure 6a). First, we introduced the *sspH* overexpression plasmid pSET152::P_hrdB_::sspH into M145 and NEAU12 to obtain strains M145/OsspH and NEAU12/OsspH, respectively. After flask fermentation, M145/OsspH exhibited a marked increase in actinorhodin production compared with the parental strain M145, while NEAU12/OsspH greatly reduced avermectin B1a production compared with NEAU12, indicating that SspH is a positive regulator of actinorhodin production in *S. coelicolor* but a repressor of avermectin biosynthesis in *S. avermitilis*. Second, genes encoding the orthologs of SspH, *sco1241* (protein product shares 72.5% sequence identity with SspH) from *S. coelicolor* and *saverm_07097* (protein product shares 74.1% sequence identity with SspH) from *S. avermitilis*, were both cloned into the integrative plasmid pSET152::P_hrdB_, generating the overexpression plasmids pSET152::P_hrdB_::1241 and pSET152::P_hrdB_::7097. The two plasmids were then introduced into *S. bingchenggensis* BC04, *S. coelicolor* M145 and *S. avermitilis* NEAU12 to obtain another six overexpression strains BC04/O1241, BC04/O7097, M145/O1241, M145/O7097, NEAU12/O1241 and NEAU12/O7097 (Figure 6a). As expected, overexpression of *sco1241* or *saverm_07097* in BC04 resulted in a significant decrease of milbemycin production; overexpression of *sco1241* or *saverm_07097* in M145 increased the titer of actinorhodin; and overexpression of *sco1241* or *saverm_07097* in NEAU12 led to marked reduction of avermectin production (Figure 6b). These results suggested that SspH and its orthologs were important regulators of antibiotic biosynthesis, sharing the same function; their effects on antibiotic production may be different from species to species.

## 3. Discussion

In recent years, with the growing emergence of drug-resistant pathogens, the occurrence of new diseases, and the demands for energy conservation, pollution reduction and high yield, the creation of new drugs and green biomanufacturing have become extremely urgent. *Streptomyces* is one of the prominent sources of natural antibiotics for drug discovery and development [27]. However, the production of most antibiotics under laboratory culture conditions is usually very low or non-existent due to tightly controlled regulatory networks, in which both global/pleiotropic and cluster-situated regulatory proteins are involved. Thus, mining new regulators and understanding their functions and molecular mechanisms in antibiotic biosynthesis is of great importance for developing regulation-based methods to increase or activate antibiotic production. In this study, a novel and highly conserved HATPase domain containing regulator, SspH, was identified to be involved in antibiotic biosynthesis in *Streptomyces*. The *sspH* overexpression could not only significantly repress milbemycin production, but also differentially influence the expression of many other potential antibiotic biosynthetic core genes. Moreover, SspH and its orthologs could also promote the high yield of actinorhodin in *S. coelicolor* and inhibit avermectin production in *S. avermitilis*, indicating that SspH and its orthologs play a universal role in controlling antibiotic production in *Streptomyces*.

Many reports have shown that global/pleiotropic regulatory protein-mediated differential control of antibiotic biosynthesis was widespread in *Streptomyces*. We took the well-studied global/pleiotropic regulators AdpA and AtrA and the quorum-sensing system as the examples. In most *Streptomyces* species, the absence of *adpA* leads to the loss of antibiotic biosynthesis, but in *Streptomyces ansochromogenes*, although *adpA* disruption led to the failure of nikkomycin production, a cryptic oviedomycin BGC was activated, leading to the detectable production of oviedomycin [28]. The regulator AtrA and its ortholog AveI have the identical functionality; they play a positive role in actinorhodin production in *S. coelicolor* and a negative role in avermectin production in *S. avermitilis*, reflecting species-differential regulation by the same regulator [29]. The quorum-sensing signal receptor ArpA mostly acts as a repressor of antibiotic production; however, some ArpA homologs also exhibit positive effects on antibiotic biosynthesis, such as *S. bingchenggensis* SbbR (positive effect on milbemycin production) [14] and *S. venezuelae* JadR3 (positive effect on jadomycin production) [30]. Therefore, the manipulation of global/pleiotropic regulatory genes has become one of the major strategies for antibiotic overproduction and activation. Here, we identified a novel and highly conserved HATPase-ol family regulator, SspH. In *S. bingchenggensis*, SspH could not only inhibit the production of milbemycins, but also promote the expression of several cryptic biosynthetic core genes. SspH and its orthologs (SCO1241 and SAVERM_07097) shared the same function; both could promote the production of actinorhodin in *S. coelicolor* but inhibited the production of avermectin in *S. avermitilis*. Thus, genetic manipulation of *sspH* and its orthologs could be applied as an effective method for future antibiotic discovery and overproduction in *Streptomyces*.

SspH has been verified to be an important regulator of antibiotic production. However, the molecular mechanism for SspH-mediated control of antibiotic biosynthesis is still not known. Luckily, detailed studies of RsbW in *Bacillus cereus* and SpoIIAB of *Bacillus subtilis* may provide some clues. Similar to SspH, RsbW and SpoIIAB are small proteins with only an HATPase domain. RsbW, an anti-σ factor possessing kinase activity, is involved in the σ^B^-mediated stress response in *Bacillus cereus*. When there was no stress, RsbW formed complexes with σ^B^ to make σ^B^ unable to interact with RNA polymerase; meanwhile, RsbW could phosphorylate a ser residue of RsbV (an anti-σ factor antagonist of RsbW), making RsbV unable to bind RsbW. Under stress, RsbV is dephosphorylated by the phosphatase so that it can form complexes with RsbW, leading to the release of σ^B^ [31]. Like RsbW, SpoIIAB from *Bacillus subtilis* is also an anti-σ factor that possesses kinase activity. SpoIIAB negatively modulates the activity of the sporulation factor σ^F^ and can phosphorylate the anti-anti-σ factor SpoIIAA. The molecular mechanism of SpoIIAB–SpoIIAA modulating the activity of σ^F^ is basically similar to that of RsbW–RsbV in the control of σ^B^ activity [32]. Here, although SspH displayed very low overall sequence similarity with RsbW and SpoIIAB, the 3D structure of SspH is highly similar to those of RsbW and SpoIIAB, thus SspH may also function by interacting with unknown proteins and by phosphorylation of certain proteins. The search for putative proteins interacting with SspH is currently under way in our laboratory, which may provide clues to improve our understanding of the HATPase-ol protein regulatory cascade in *Streptomyces*.

## 4. Materials and Methods

### 4.1. Strains, Plasmids and Culture Conditions

All strains used in this work were listed in Table S6. *S. bingchenggensis* BC04 is an industrial milbemycin producer generated from *S. bingchenggensis* CGMCC 1734 after random mutagenesis (GenBank Accession No. CP002047.1) [12]. *S. coelicolor* M145 is a prototrophic derivative of strain *S. coelicolor* A3(2) lacking the endogenous plasmid SCP1 and SCP2 (GenBank Accession No. AL645882.2) [33]. *S. avermitilis* NEAU12 is an avermectin B1a producing strain, and its genome shares high similarity with the genome sequence of *S. avermitilis* MA-4680 (GenBank Accession No. NC_003155.5) [34]. All *E. coli* strains were grown on Luria–Bertani (LB) medium supplemented with antibiotics as required at 37 °C [35]. For conjugation, *E. coli* ET12567 (pUZ8002) was used for transferring plasmids from *E. coli* to *Streptomyces*, and *Streptomyces* strains were grown on MS agar at 28 °C [36]. For spore collection, *S. bingchenggensis*, *S. coelicolor*, *S. avermitilis* and their derivatives were grown at 28 °C on SKYM agar plates [12] and MS agar plates [36,37], respectively. Flask fermentation for the production of milbemycins, actinorhodin and avermectins were the same as previously reported [12,36,37].

All plasmids and primers used in this work were listed in Tables S7 and S8, respectively. A quantity of pSET152, which can integrate into the *Streptomyces* chromosome by site-specific recombination at the phage ΦC31, was used to create recombinant plasmids for overexpressed mutant strains [38]. Meanwhile, pSETddCpf1 was used to construct plasmids for CRISPRi gene suppression mutant strains [39]. Additionally, pBluescript KS (+) was used for site-directed mutagenesis as previously reported [12].

### 4.2. Gene Overexpression and Repression

For gene overexpression, three fragments containing the coding region of *sspH*, *sco1241* and *saverm_7097* were individually amplified from the genomic DNAs of *S. bingchenggensis* BC04, *S*. *coelicolor* M145 and *S. avermitilis* NEAU12 using primers SspH-F/R, sco1241-F/R and saverm_7097-F/R. The three fragments were then cloned into the EcoRI/XbaI sites of pSET152::P_hrdB_ to generate gene overexpression plasmids pSET152::P_hrdB_::sspH, pSET152::P_hrdB_::1241 and pSET152::P_hrdB_::7097, respectively. The three plasmids were separately introduced into three strains including *S.* *bingchenggensis* BC04, *S*. *coelicolor* M145 and *S. avermitilis* NEAU12, and nine overexpression strains BC04/OsspH, BC04/O1241, BC04/O7097, M145/OsspH, M145/O1241, M145/O7097, NEAU12/OsspH, NEAU12/O1241 and NEAU12/O7097 were obtained (Table S7).

The *sspH* repression strain was constructed based on pSETddCpf1 [39]. Meanwhile, *sspH* specific crRNA expression cassette was amplified using pSETddCpf1 as the template, and ddCpf1-sspH-F and the ddCpf1-R (crRNA-rev) as the primer pair. Additionally, *sspH* specific crRNA cassette was digested with NdeI/SpeI and ligated into the NdeI/SpeI sites of pSETddCpf1, generating *sspH* repression plasmid pSETddCpf1::sspH. Next, pSETddCpf1::sspH was introduced into *S. bingchenggensis* BC04 to obtain the repression strain BC04/RsspH.

### 4.3. Site-Directed Mutagenesis of sspHpH

In order to construct the *sspH* mutant plasmids, the fragment containing the constitutive promoter P*_hrdB_* and *sspH* coding region was amplified using pSET152::P_hrdB_::sspH as the template. This fragment was then cloned into the EcoRI/BamHI sites of pBluescriptKS (+) to obtain pBluescriptKS (+)::P_hrdB_::sspH, which was used as the template to conduct subsequent site-directed mutation experiments. The primers for site-directed mutagenesis were phosphorylated at the 5′ end with T4 polynucleotide kinase, respectively, and were further used for amplification of mutant *sspH*. The PCR products were purified by gel extraction and ligated by self-ligation to generate mutagenized *sspH* plasmids. The SspH mutants were N54A (AAC to GCC) and D84A (GAC to GCC). Finally, the DNA fragments containing mutant *sspH* were cut from pBluescriptKS (+) and ligated into the EcoRI/BamHI sites of pSET152, generating pSET152::P_hrdB_::N54A and pSET152::P_hrdB_::D84A, respectively. Subsequently, the two plasmids were integrated into BC04 to obtain the *sspH* mutant overexpression strains BC04/N54A and BC04/D84A.

### 4.4. Detection Methods of Milbemycins, Actinorhodin and Avermectins

For milbemycin A3/A4 and avermectin B1a analysis, 0.5 mL fermented cell cultures of *S. bingchenggensis* was mixed with three volumes of ethanol to extract milbemycin A3/A4, and 0.5 mL fermentation cultures of *S. avermitilis* was mixed with three volumes of methanol. HPLC analysis was carried out by Agilent 1260 II system with an SB-C18 column (Zorbax, 4.6 mm × 260 mm, 5 mm) at a flow rate of 1.0 mL/min. For milbemycin A3/A4, a linear gradient of solvent B was applied from 0 to 100% in 15 min (Solvent A: CH_3_CN:H_2_O:CH_3_OH = 350:50:100, *v*/*v*/*v*; Solvent B: CH_3_OH), and samples were detected at 242 nm. For avermectin B1a, samples were detected at 246 nm with 90% methyl alcohol. For the detection of actinorhodin, 0.5 mL of fermented cell cultures was treated with 0.5 mL NaOH (1 M NaOH), centrifuged, and the OD_608nm_ of the supernatant was determined [40].

### 4.5. RNA Isolation and Quantitative Real-Time PCR (qRT-PCR) Assay

RNAs were isolated from the fermentation cultures of *S. bingchenggensis* grown at 28 °C at different time points (2, 3, 4, 6 and 8 days). The detailed steps for RNA extraction were described as previously [12]. Each RNA sample was treated with RQ1 RNase-free DNase I (Promega) to exclude the possibility of genomic DNA contamination. UV spectroscopy and agarose gel electrophoresis were used to detect the RNA quality and quantity. For qRT-PCR, 1 μg of total RNAs was used for first-strand cDNA synthesis, and primers were designed to amplify fragments of 100–200 bp (Table S8). The PCR procedures were the same as previously reported [12]. After PCR amplification, the data were analyzed using LightCycler^®^-96 Series Software (v1.4.1, Roche Diagnostics, Rotkreuz, Switzerland) and the 2^−ΔΔCT^ Method [41].

### 4.6. Sequence Analysis

Protein sequence alignment and domain architectures were analyzed by publicly available databases and their application tools, including BLAST (http://www.blast.ncbi.nlm.nih.gov/Blast.cgi, accessed on 18 June 2021) and SMART (http://www.smart.embl-heidelberg.de/, accessed on 18 June 2021), KEGG (http://www.genome.jp/kegg/, accessed on 18 June 2021).

### 4.7. Statistical Analysis

All experiments were run in three biological triplicates independently. Data were presented as averages of three triplicates. Significance was analyzed by Student’s *t*-test (GraphPad Prism 6), and the significance were presented as follows, * *p* < 0.05, ** *p* < 0.01, *** *p* < 0.001, and no marked means no significant.

### 4.8. Data Availability

The transcriptomic data of *S. bingchenggensis* BC04 collected on day 3 can be found in GEO database (GSE147644).

## 5. Conclusions

In this work, we identified a new and highly conserved regulator of antibiotic biosynthesis, SspH, in *Streptomyces*. SspH is a small and single domain protein with only the HATPase domain. Overexpression of *sspH* repressed the transcription of milbemycin biosynthetic genes and consequently decreased milbemycin production. The *sspH* overexpression also differentially influenced the expression of many other antibiotic biosynthetic core genes present in the genome of *S. bingchenggensis*. Moreover, SspH and its orthologs from *S. coelicolor* and *S. avermitilis* shared the same function, and all could promote the production of actinorhodin and inhibit the production of avermectin. These results extended our understanding of the regulatory network of antibiotic biosynthesis, provided effective targets for antibiotic discovery and overproduction, and also provided a useful method to mine new antibiotic regulators.

## Figures and Tables

**Table 1:** 11 candidate transcriptional regulatory genes with high FPKM values (FPKM > 2000) on day 3.

Gene ID	Type of Product	Reported Homolog	Predicted Function	Reference(s)
*sbi_05734*	MerR	BldC (SCO4091)	Widespread; a repressor to maintain vegetative growth and to delay entry into development.	[17]
*sbi_06842*	SARP	-	A cluster-situated activator of type II PKS cluster, also essential for milbemycin production.	[15]
*sbi_03959*	Wbl	WblE (SAVERM_3016)	Widespread; an important regulator of morphological differentiation.	[18]
*sbi_05953*	Lsr2	Lsr2 (SVEN_3225)	Widespread; a nucleoid-associated protein that influences chromosome segregation, DNA replication, transcription and secondary metabolism.	[19]
*sbi_03799*	TCS (RR)	SCO5351	Widespread; a pleiotropic regulator involved in secondary metabolism and development.	[20]
*sbi_06268*	TCS (RR)	BldM (SCO4768)	Widespread; an essential regulator for aerial hyphae formation.	[21]
*sbi_08867*	HATPase-ol	-	Widespread; function unknown.	This work
*sbi_03479*	TCS (HK)	SbrK	A repressor of milbemycin biosynthesis.	[16]
*sbi_05811*	Wbl	WblA (SCO3579)	Widespread; a global regulator involved in differentiation and secondary metabolism.	[22]
*sbi_05271*	MarR	-	Function unknown.	-
*sbi_04164*	Wbl	WblI (SCO5046)	Widespread; a positive impact on antibiotic production.	[23]

## Data Availability

The datasets supporting the conclusions of this article are included within the article and the Supplementary Materials.

## Supplementary Materials

The following supporting information can be downloaded at: https://www.mdpi.com/article/10.3390/antibiotics11050538/s1, Figure S1: Secondary and three-dimensional (3D) structure of SspH, Figure S2: Amino acid sequence alignment of SspH and its orthologs, Table S1: Genes whose products contain only the HATPase domain in several *Streptomyces* species, Table S2: Amino acid sequences alignment of homologous proteins from *S*. *bingchenggensis* and other *Streptomyces* species, Table S3: Number of hits of SspH homologous sequence in different bacteria after PSI-BLAST search, Table S4: Three milbemycin biosynthetic genes (*milR*, *milF* and *milA1*) and their functions, Table S5: 31 antibiotic biosynthetic core genes and their putative functions, Table S6: Strains used in this work, Table S7: Plasmids used in this work, Table S8: Primers used in this work.
