# Supplementary material for: SspH, a Novel HATPase Family Regulator, Controls Antibiotic Biosynthesis in Streptomyces
Source: Antibiotics (Basel). 2022 Apr 19;11(5):538. doi: 10.3390/antibiotics11050538 (PMC9137472; doi:10.3390/antibiotics11050538)
Supplement: Supplementary file 1 [file antibiotics-11-00538-s001.zip › antibiotics-1681649-supplementary.pdf]

Supplementary Materials

SspH, a novel HATPase family regulator, controls antibiotic biosynthesis in *Streptomyces*

Xue Yang [1,†], Yanyan Zhang [1,†,*], Shanshan Li [1], Lan Ye [1,2], Xiangjing Wang [2], Wensheng Xiang [1,2,*]

[1] State Key Laboratory for Biology of Plant Diseases and Insect Pests, Institute of Plant Protection, Chinese Academy of Agricultural Sciences, No. 2 Yuanmingyuan West Road, Haidian District, Beijing, 100193, China; yangxue504499816@163.com (X.Y.); ssli@ippcaas.cn (S.L.); yelan_617@163.com (L.Y.)

[2] School of Life Science, Northeast Agricultural University, No. 59 Mucai Street, Xiangfang District, Harbin 150030, China; wangneau2013@163.com (X.W.)

* Correspondence: yyzhang@ippcaas.cn (Y.Z.), xiangwensheng@neau.edu.cn (W.X.)

† These authors contributed equally to this work.

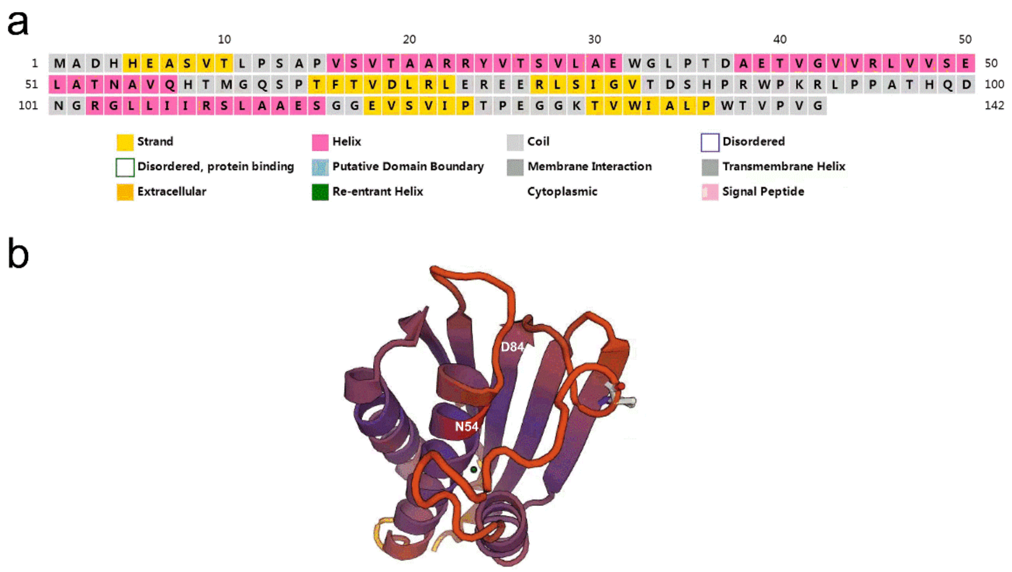

Figure S1. Secondary and three-dimensional (3D) structure of SspH. (**a**) Secondary structure of SspH. (**b**) Putative 3D structure. The homologous three - dimensional (3D) structure model for SspH was constructed based on the *Bacillus* anti - sigma factor SpoIIAB.

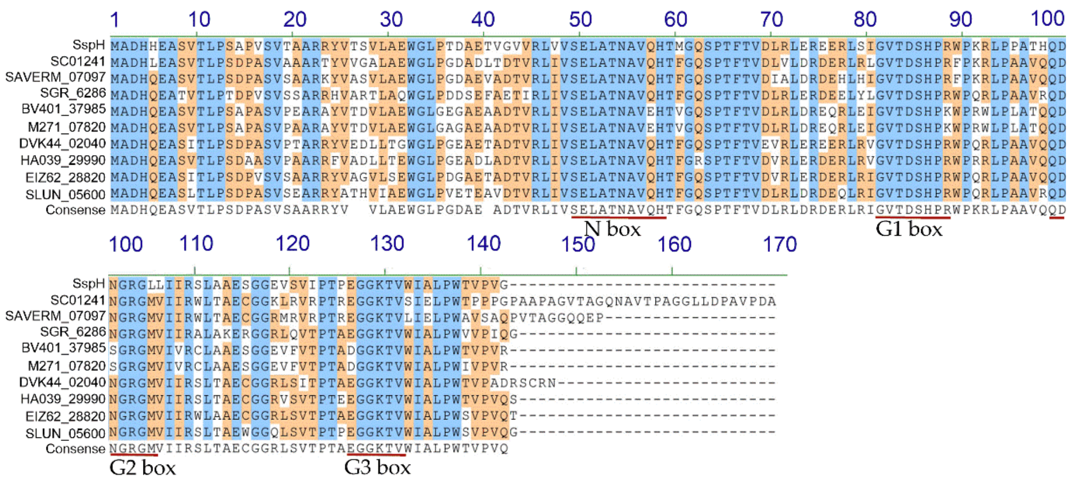

Figure S2. Amino acid sequence alignment of SspH and its orthologs. Identical or similar residues in all sequences are highlighted in blue and orange, respectively. The corresponding assumed conserved motifs are marked in red line. SCO1241, SspH ortholog from *S. coelicolor*; SAVERM_7097, SspH ortholog from *S. avermitilis*; SGR_6286, SspH ortholog from *S. griseus*, BV401_37985, SspH ortholog from *S. autolyticus*; M271_07820, SspH ortholog from *S. rapamycinicus*; DKV44_02040, SspH ortholog from *S. paludis*, HA039_29990, SspH ortholog from *S. liangshanensis*; EIZ62_28820, SspH ortholog from *S. ficellus*; SLUN_05600, SspH ortholog from *S. lunaelactis*.

Table S1. Genes whose products contain only the HATPase domain in several *Streptomyces* species

Strain	Gene ID
S. coelicolor	*sco0673, sco0702, sco0868, sco1241, sco2864, sco3066, sco3423, sco3724, sco3930, sco4412, sco4677, sco4941, sco5103, sco5460, sco5915, sco5978, sco6130, sco6156, sco6237, sco6749, sco6949, sco7313, sco7328, sco7446, sco7328, sco7614*
S. avermitilis	*saverm_0234, saverm_1437, saverm_1664, saverm_2076, saverm_2174, saverm_2785, saverm_2797, saverm_3186, saverm_3424, saverm_3488, saverm_3738, saverm_3761, saverm_3991, saverm_4086, saverm_4225, saverm_4398, saverm_4615, saverm_4783, saverm_5014, saverm_5201, saverm_5409, saverm_5755, saverm_6252, saverm_7097*
S. bingchenggensis	*sbi_02080, sbi_02469, sbi_02795, sbi_03226, sbi_03585, sbi_03656, sbi_03703, sbi_03978, sbi_04142, sbi_04846, sbi_05023, sbi_05275, sbi_05655, sbi_05899, sbi_06283, sbi_06400, sbi_06722, sbi_07227, sbi_08076, sbi_08867, sbi_09291*

Table S2. Amino acid sequences alignment of homologous proteins from *S. bingchenggensis* and other *Streptomyces* species

Proteins	Coverage/identity(%) to genes of
SBI_05734	*S. coelicolor*; BldC (SCO4091): 100/100
SBI_03959	*S. avermitilis*; WblE (SAVERM_3016): 78/100
SBI_05953	*S. venezuelae*; Lsr2 (SVEN_3225): 99/66
SBI_03799	*S. coelicolor*; SCO5351: 84/81
SBI_06268	*S. coelicolor*; BldM (SCO4768): 100/100
SBI_05811	*S. coelicolor*; WblA (SCO3579): 100/89
SBI_04164	*S. coelicolor*; WblI (SCO5046): 76/86

Table S3. Number of hits of SspH homologous sequence in different bacteria after PSI-BLAST search

Organism	Number of hits
Streptomyces	1379
Kitasatospora	3
Streptosporangium nondiastaticum	2
Actinospica acidiphila	6
unclassified Actinomycetia	4
Mesorhizobium sp. B2-3-3 (non-Actinomycetia)	1

Table S4. Three milbemycin biosynthetic genes (*milR*, *milF* and *milA1*) and their functions

Gene ID	Type of product	Function	Reference
sbi_00734 (*milR*)	MilR	LAL family regulator; a cluster-situated key activator of milbemycin biosynthesis; activating the transcription of the *milA4-E* operon and *milF* directly and activating the transcription of the other milbemycin biosynthetic structural genes indirectly.	[1]
sbi_00731 (*milF*)	MilF	The tailoring enzyme gene encoding C5-ketoreductase responsible for the ketonization of milbemycins.	[2]
sbi_00789 (*milA1*)	MilA1	The milbemycin polyketide synthase (PKS) gene; containing the loading module and two extension modules responsible for polyketide chain extension.	[3]

Table S5. 31 antibiotic biosynthetic core genes and their putative functions

Biosynthetic core gene	Putative function	Predicted product type
sbi_00140	amino acid adenylation domain-containing protein	PKS-NRPS hybrid for moenomycin
sbi_00220	putative squalene-hopene cyclase	Terpene
sbi_00319	modular polyketide synthase	PKS-NRPS hybrid for leinamycin
sbi_00522	modular polyketide synthase	PKS-NRPS hybrid for BE-14106
sbi_00625	amino acid adenylation domain-containing protein	NRPS
sbi_00655	modular polyketide synthase	Type I PKS for pimaricin
sbi_00671	putative mixed polyketide synthase/non-ribosomal peptide synthetase	Type I PKS for meridamycin
sbi_00822	non-ribosomal peptide synthetase	NRPS
sbi_00956	non-ribosomal peptide synthetase	PKS-NRPS hybrid
sbi_01029	putative polyketide synthase	Type I PKS
sbi_01080	pentalenene synthase	Terpene
sbi_01365	amino acid adenylation domain-containing protein	PKS-NRPS hybrid for lasalocid
sbi_01540	putative polyketide synthase	Type I PKS for borrelidin
sbi_01983	modular polyketide synthase	Type I PKS for mycinamicin
sbi_02068	putative germacradienol synthase	Terpene
sbi_02232	group-specific protein	Cyclothiazomycin
sbi_02590	squalene/phytoene synthase	Terpene for hopanoid
sbi_02764	3-oxoacyl-ACP synthase I	Type II PKS for grey spore pigment
sbi_02988	amino acid adenylation domain protein	PKS-NRPS for calcium-dep antibiotic

Biosynthetic core gene	Putative functions	Predicted product type
sbi_03179	type I modular polyketide synthase	PKS-NRPS hybrid for borrelidin
sbi_06052	amino acid adenylation domain-containing protein	PKS-NRPS hybrid
sbi_06449	non-ribosomal peptide synthetase	Desferrioxamin
sbi_06799	non-ribosomal peptide synthetase	NRPS for melanin
sbi_06873	glycosyl transferase	Type II PKS for Polyketomycin
sbi_08414	modular polyketide synthase	Nanchangmycin (by type I PKS)
sbi_08957	2-methylisoborneol synthase	Terpene
sbi_09057	putative non-ribosomal peptide synthase	Melanin
sbi_09195	modular polyketide synthase	PKS-NRPS hybrid for lymphostin
sbi_09249	non-ribosomal peptide synthetase	NRPS for moenomycin
sbi_09652	non-ribosomal peptide synthetase	Oxazolomycin (by PKS-NRPS hybrid)
sbi_10015	putative type I polyketide synthase WcbR	Type I PKS

Table S6. Strains used in this work

Strain	Characteristics	Source
Escherichia coli		
E. coli JM109	Host for all plasmids cloning procedures	Novagen
ET12567 (pUZ8002)	Donor strain for conjugation between *E. coli* and *Streptomycetes*	[1]
Streptomyces bingchenggensis		
BC04	Milbemycin high-yield strain obtained by random mutagenesis	[4]
BC04-C1	BC04 carrying pSET152	This work
BC04-C2	BC04 carrying pSETddCpf1	This work
BC04/OsspH	BC04 carrying pSET152::P_{hrdB}::sspH	This work
BC04/RsspH	BC04 carrying pSETddCpf1::sspH	This work
BC04/O1241	BC04 carrying pSET152::P_{hrdB}::1241	This work
BC04/O7097	BC04 carrying pSET152::P_{hrdB}::7097	This work
Streptomyces coelicolor		
M145	Actinorhodin producer	[1]
M145/OsspH	M145 carrying pSET152::P_{hrdB}::sspH	This work
M145/O1241	M145 carrying pSET152::P_{hrdB}::1241	This work
M145/O7097	M145 carrying pSET152::P_{hrdB}::7097	This work
Streptomyces avermitilis		
NEAU12	Avermectin high-yield producer	Laboratory storage
NEAU12/OsspH	NEAU12 carrying pSET152::P_{hrdB}::sspH	This work
NEAU12/O1241	NEAU12 carrying pSET152::P_{hrdB}::1241	This work
NEAU12/O7097	NEAU12 carrying pSET152::P_{hrdB}::7097	This work

Table S7. Plasmids used in this work

Names	Characteristics	Source
pSET152	Apr^R, integrative *E. coli-Streptomyces* shuttle vector	[1]
pSET152::P_{hrdB}	pSET152 containing P_{hrdB}	This work
pSET152::P_{hrdB}::sspH	pSET152::P_{hrdB} containing *sspH*	This work
pSET152::P_{hrdB}::1241	pSET152::P_{hrdB} containing *sco1241*	This work
pSET152::P_{hrdB}::7097	pSET152::P_{hrdB} containing *saverm_7097*	This work
pSETddCpf1	pSET152 with the scocpf1 (E1006A) gene under the control of the *ermE*p* promoter	[5]
pSETddCpf1::sspH	pSETddCpf1 containing the crRNA transcription cassette for editing *sspH*	This work
pBluescript II KS (+)	Amp^R, *E. coli* cloning vector	Stratagene
pBluescript II KS(+)::P_{hrdB}::sspH	pBluescript II KS(+) containing *sspH* under the control of P_{hrdB}	This work
pBluescript II KS(+)::P_{hrdB}::N54A	pBluescript II KS(+) containing mutagenized *sspH* (mutation of Asn54 to Ala) under the control of P_{hrdB}	This work
pBluescript II KS(+)::P_{hrdB}::D84A	pBluescript II KS(+) containing mutagenized *sspH* (mutation of Asp84 to Ala) under the control of P_{hrdB}	This work
pSET152::P_{hrdB}::N54A	pSET152 containing mutagenized *sspH* (mutation of Asn54 to Ala) under the control of P_{hrdB}	This work
pSET152::P_{hrdB}::D84A	pSET152 containing mutagenized *sspH* (mutation of Asp84 to Ala) under the control of P_{hrdB}	This work

Table S8. Primers used in this work

Name	Sequence (5′-3′)	Usage
sspH-F	GCCGTTTTTCAACGTTCCGAGAGGTTGTTCATGGCTGACCACCACGAAGCAT	Construction of pSET152::P_{hrdB}::sspH
sspH-R	CGACTCTAGAGGATCCGCGGCCGCGCGATCACCACGGGGCACGTCAAGC	
ddCpf1-sspH-F	GACTAGTCGTTCCAGCCGCAGATCCACCGTATCTACAACAGTAGAAATTTGG	Construction of pSETddCpf1::sspH
ddCpf1-R	GGAATTCCATATGTGGATCCTACCAACCGGCACGATT	
sco1241-F	GCCGTTTTTCAACGTTCCGAGAGGTTGTTCATGGCAGACCATCTGGAAGCATCC	Construction of pSET152::P_{hrdB}::1241
sco1241-R	CGACTCTAGAGGATCCGCGGCCGCGCGATAAGGGCCGGATCTGGCACGC	
saverm_7097-F	GCCGTTTTTCAACGTTCCGAGAGGTTGTTCATGGCAGATCACCAGGAAGCATCCG	Construction of pSET152::P_{hrdB}::7097
saverm_7097-R	CGACTCTAGAGGATCCGCGGCCGCGCGATTCACCCGGACGGCCGAACACC	
N54A-F	GCCGCCGTCCAGCACACGATGGGCCAGTCGCC	Construction of pSET152::P_{hrdB}::D54A
N54A-R	GGTGGCGAGTTCGGAGACGACGAGCCGTACGA	
D84A-F	GCCAGCCACCCCAGATGGCCCAAACGCCTGCC	Construction of pSET152::P_{hrdB}::D84A
D84A-R	GGTGACGCCGATGCTCAGCCGCTCTTCGCGTT	

Name	Sequence (5′-3′)	Usage
Q16S-F	TGTCGTGAGATGTTGGGTTAAG	qRT-PCR analysis of 16S rRNA
Q16S-R	TCATTGTACCGGCCATTGTAG	
QmilA1-F	GCCGTATCGTCTTCGTCTTC	qRT-PCR analysis of *milA1*
QmilA1-R	TGGAGGATGTCCGTGAGT	
QmilR-F	GCAAGAGCGAATTCCTGAAG	qRT-PCR analysis of *milR*
QmilR-R	CTGTGACGGAGCGAGTC	
QmilF-F	AGGTCACTCAGGAGGAGTT	qRT-PCR analysis of *milF*
QmilF-R	GAGGAGATGTTGACGATCCG	
QsspH-F	GGCTGGAACGCGAAGAG	qRT-PCR analysis of *sspH*
QsspH-R	CCGACCGTTGTCCTGATG	
Qsbi_00140-F	GGGAGCGTGTCGAAGAAG	qRT-PCR analysis of *sbi_00140*
Qsbi_00140-R	GCACACCGAGTGATGTATGT	
Qsbi_00220-F	CTGTCCGTCTTCTGCTATGTC	qRT-PCR analysis of *sbi_00220*
Qsbi_00220-R	CGAGCATGGTCGGATTGT	
Qsbi_00319-F	TGTACGTCAACACCACCAC	qRT-PCR analysis of *sbi_00319*
Qsbi_00319-R	AGTACGGCGTGCACATT	
Qsbi_00522-F	AAAGGCACGATCCGTACAC	qRT-PCR analysis of *sbi_00522*
Qsbi_00522-R	GGCCTCTTTCGGCGATATAC	
Qsbi_00625-F	CTTCGCCAACTACTTCCACTC	qRT-PCR analysis of *sbi_00625*
Qsbi_00625-R	CCATGAACATGTCGAAGGAGAA	

Name	Sequence (5′-3′)	Usage
Qsbi_00655-F	AGCTACCAGGACTACACCA	qRT-PCR analysis of *sbi_00655*
Qsbi_00655-R	CTCGAGCCCGAACACATAG	
Qsbi_00671-F	GGCGTGATGAGCAACGATTA	qRT-PCR analysis of *sbi_00671*
Qsbi_00671-R	CCGGTGAAGTCGAAGAAGTG	
Qsbi_00822-F	CTGCCGCTGTTTCCCTATATC	qRT-PCR analysis of *sbi_00822*
Qsbi_00822-R	AATTCATCGGGCCGGTTC	
Qsbi_00956-F	CACCGTACGTAACGCGAAAT	qRT-PCR analysis of *sbi_00956*
Qsbi_00956-R	CGTAGAGGTCTGTTGCGTATTC	
Qsbi_01029-F	CACCTGGTGTACGTCGAAAT	qRT-PCR analysis of *sbi_01029*
Qsbi_01029-R	AGGCCGTCCATGCTTTC	
Qsbi_01080-F	ACGGAGTATCTGCACTCCTA	qRT-PCR analysis of *sbi_01080*
Qsbi_01080-R	CGAACCGCTCGTTGAGAT	
Qsbi_01365-F	GAGAGATCGAGGAGTGGGAA	qRT-PCR analysis of *sbi_01365*
Qsbi_01365-R	GGTCGAAAGCGGTCTTGT	
Qsbi_01540-F	CTGTGGAGTTGCTGGATTCT	qRT-PCR analysis of *sbi_01540*
Qsbi_01540-R	CGTCCTCCAACGACCAATC	
Qsbi_01983-F	GACTACTGGTACCGCAATCTG	qRT-PCR analysis of *sbi_01983*
Qsbi_01983-R	CGAGCTGACCTCGATGAAC	
Qsbi_02068-F	TTCTTCTTCGACGACCACTTC	qRT-PCR analysis of *sbi_02068*
Qsbi_02068-R	GGGAGCACCGGCATAAA	

Name	Sequence (5′-3′)	Usage
Qsbi_02232-F	CCGCTTCGAGCGCTATC	qRT-PCR analysis of *sbi_02232*
Qsbi_02232-R	GTGAGCACCTGTACCTGAC	
Qsbi_02590-F	CATGGCGATCTACGGCTAC	qRT-PCR analysis of *sbi_02590*
Qsbi_02590-R	AGCATCATGAGCGGATCG	
Qsbi_02764-F	TGGAGCAGGACTACGTCAA	qRT-PCR analysis of *sbi_02764*
Qsbi_02764-R	GAAGGCGTGGTGCAGATAC	
Qsbi_02988-F	CATGAGCTGCTGGCATATCT	qRT-PCR analysis of *sbi_02988*
Qsbi_02988-R	ATGTACAGCGGCAGTGATG	
Qsbi_03179-F	CCATTCGTCCTTCTGGGATATT	qRT-PCR analysis of *sbi_03179*
Qsbi_03179-R	GGACTTGGTGTTCCGTCTC	
Qsbi_06052-F	TCCGGCGTATCTCATCTACA	qRT-PCR analysis of *sbi_06052*
Qsbi_06052-R	GTGACATCAGGAGGAAGGTC	
Qsbi_06449-F	GTCCTTTCGGGCGGATTT	qRT-PCR analysis of *sbi_06449*
Qsbi_06449-R	GCTGAAGCGTCAGAGAGTAAG	
Qsbi_06799-F	TCCTGGTCTTCGAGAACTATCC	qRT-PCR analysis of *sbi_06799*
Qsbi_06799-R	TAGTGGGTGGCGTCCTT	
Qsbi_06873-F	CTGGACCGGCAGGATCT	qRT-PCR analysis of *sbi_06873*
Qsbi_06873-R	CACAGGTCGGGCTCGTA	
Qsbi_08414-F	ACGCCTCCTTCTTCAACATC	qRT-PCR analysis of *sbi_08414*
Qsbi_08414-R	GGTGAGGGTGCCTTTGAG	

Name	Sequence (5′-3′)	Usage
Qsbi_08957-F	CTGTACTCGTACACCAAGGAAC	qRT-PCR analysis of *sbi_08957*
Qsbi_08957-R	TCGACCGCCTTCAGGTA	
Qsbi_09057-F	GCGTACTGAACACCGAGAAG	qRT-PCR analysis of *sbi_09057*
Qsbi_09057-R	GATCTGAGTCTCCAGTTTGAGC	
Qsbi_09195-F	GCCTGTACGAGAGTGGTTATC	qRT-PCR analysis of *sbi_09195*
Qsbi_09195-R	CGCAGATCCAGTGTGTAGG	
Qsbi_09249-F	CACCTTCTACCACCGGAAAG	qRT-PCR analysis of *sbi_09249*
Qsbi_09249-R	AAAGTCGGAGAACGGTGTG	
Qsbi_09652-F	CGTCATGAACAACGACTACGA	qRT-PCR analysis of *sbi_09652*
Qsbi_09652-R	AATCGAGCACATACGACACC	
Qsbi_10015-F	GACTATGCCTTCCACAGCAA	qRT-PCR analysis of *sbi_10015*
Qsbi_10015-R	CCTGTGACGGTGGAGTAGA	

References

1. Zhang, Y.; He, H.; Liu, H.; Wang, H.; Wang, X.; Xiang, W. Characterization of a pathway-specific activator of milbemycin biosynthesis and improved milbemycin production by its overexpression in *Streptomyces bingchenggensis*. *Microb. Cell Fact.* **2016**, *15*, 152.
2. Wang, H.; Zhang, J.; Zhang, Y.; Zhang, B.; Liu, C.; He, H.; Wang, X.; Xiang, W. Combined application of plasma mutagenesis and gene engineering leads to 5-oxomilbemycins A3/A4 as main components from *Streptomyces bingchenggensis*. *Appl. Microbiol. Biotechnol.* **2014,** *98*, 9703–9712.
3. Kim, M.S.; Cho, W.J.; Song, M.C.; Park, S.W.; Kim, K.; Kim, E.; Lee, N.; Nam, S.J.; Oh, K.H.; Yoon, Y.J. Engineered biosynthesis of milbemycins in the avermectin high-producing strain *Streptomyces avermitilis*. *Microb. Cell Fact*. **2017**, *16*, 9.
4. Keiser, T.; Bipp, M.; Buttner, M.; Chater, K.F.; Hopwood, D.A. *Practical Streptomyces Genetics*. The John Innes Foundation, Norwich, UK, 2000.
5. Li, L.; Wei, K.; Zheng, G.; Liu, X.; Chen, S.; Jiang, W.; Lu, Y. CRISPR-Cpf1-Assisted Multiplex Genome Editing and Transcriptional Repression in *Streptomyces*. *Appl. Environ. Microbiol.* **2018**, *84*, e00827-18.
